# A simple approach for the preoperative assessment of sacral morphology for percutaneous SI screw fixation

**DOI:** 10.1007/s00402-016-2528-3

**Published:** 2016-08-06

**Authors:** Michael Goetzen, Kevin Ortner, Richard A. Lindtner, Rene Schmid, Michael Blauth, Dietmar Krappinger

**Affiliations:** Department of Trauma Surgery, Medical University of Innsbruck, Anichstraße 35, 6020 Innsbruck, Austria

**Keywords:** Sacral morphology, SI screw fixation, Sacral corridor, Pelvic ring, Pelvic ring fracture

## Abstract

**Background:**

Percutaneous sacroiliac screw fixation under fluoroscopic control is an effective method for posterior pelvic ring stabilization. However, sacral dysmorphism has a high risk of L5 nerve injury. This study describes a simple method for the preoperative assessment of the sacral morphology using CT scans with widely available tools.

**Materials and methods:**

CT scans of 1000 patients were analyzed. True inlet, outlet, and lateral views of the sacrum were obtained using a two-dimensional reconstruction tool to align the sacrum in a reproducible manner. Corridor morphology in the inlet view was measured to calculate different morphological types: (1) Ascending type, (2) Horizontal type, and (3) Descending type. In a second step, the corridor was analyzed for the presence of an anterior indentation of the sacrum between the SI joint and the midsagittal plane with proximity to the nerve root L5, which, therefore, may be harmed during screw misplacement.

**Results:**

A notch was found in the majority of cases with relative frequencies ranging from 69 % (upper quartile of S1) to 95 % (upper quartile of S2). Descending types were, by far, the most frequent corridor type with one exception: In the upper quartile of S1, the ascending type was the most frequent corridor (71 %). Horizontal types were less frequent with a relative incidence between 2 and 14 %.

**Discussion:**

This study should increase the awareness for sacral dysmorphism, emphasize the importance of a preoperative assessment of the osseous corridor, and provide a simple method for the preoperative assessment with widely available tools.

## Introduction

Percutaneous sacroiliac (SI) screw fixation is a minimally invasive and effective method for the stabilization of the posterior pelvic ring [1–6]. Screw placement under two-dimensional (2D) fluoroscopic control is the gold standard [4, 6–8]. CT-controlled and navigated SI screw fixation techniques are demanding procedures and, therefore, not widely available beyond trauma centers [6, 9–15].

Major complications of fluoroscopically controlled percutaneous SI screw fixation include nerve and vessel lesions following screw misplacement. The relative risk of lesions of the fifth lumbar nerve (L5), for example, is up to 8 % [6, 16–20]. Variations in the morphology of the upper sacrum, widely known as ‘‘sacral dysmorphism’’, [21, 22] primarily account for this relatively high complication rate. Three-dimensional (3D) models of the upper sacrum have been described in the literature to assess safe corridors for SI screw placement even in the presence of sacral dysmorphism [23–25]. These techniques, however, require particular planning software and are typically not applicable to the surgeon during preoperative assessment.

The aim of this study, therefore, was to present a simple method for the preoperative assessment of the sacral morphology using CT scans with widely available tools. In addition, it was our goal to classify the morphology of the upper sacrum based on the risk of iatrogenic nerve lesions during percutaneous SI screw fixation.

## Materials and methods

CT scans of a consecutive series of 1000 patients were analyzed in this study. The patients were included irrespective of gender, health conditions, or premorbidities. Exclusion criteria were:

1. Age <18 years.
2. Recent or consolidated pelvic fractures.
3. Osteolytic pelvic lesions.

All patients underwent abdominal CT scans, which include the osseous pelvis, in a supine position with hip and knee joints extended. CT scans were performed during clinical routine. Indications for the CT scans were different from the aim of this study. Accordingly, the patients were not exposed to additional radiation for the purpose of this study. A 64-section CT scanner (LightSpeed VCT, GE Healthcare, Chalfont St. Giles, UK) was used to perform the CT scans. The CT images were taken with 0.6 mm slice depth and saved in slices of 1.25 mm in thickness. 2D reconstructions and measurements were performed with both a standard picture archiving and communication system (PACS) and software (Impax EE R20 XIV SU2, AGFA diagnostic software, Ridgefield Park, NJ).

**Fig. 1:**
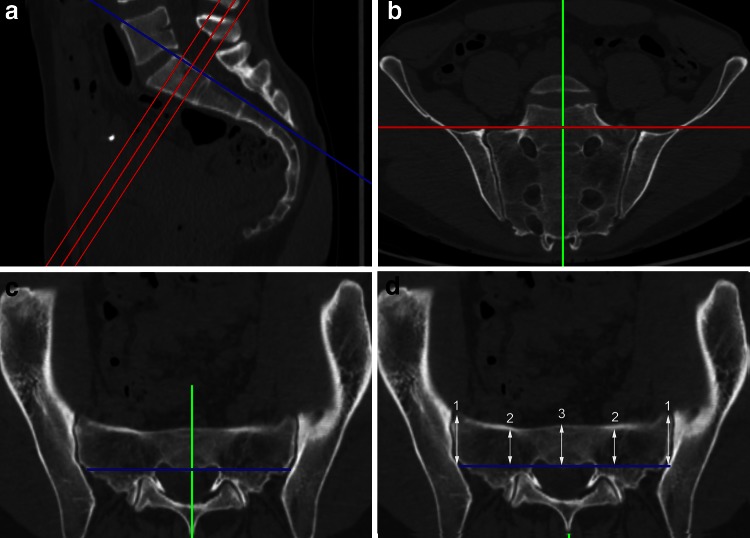
**a** Alignment in the *lateral view* in a midsagittal plane: parallel to the lower endplate of S1 in the upper, middle, and lower quartiles of the body S1 (*red lines*) and along the posterior boarder of S1 (*blue line*). **b** Alignment in the *outlet view*: along the vertical axis of the sacrum (*green line*) and parallel to a line connecting the upper borders of the SI joint (*red line*). **c** Alignment in the *inlet view*: from the middle of the promontorium to the spinous process (*green line*) and parallel to the posterior wall of the body of S1 (*blue line*). **d** Assessment of the width of the corridor in the *inlet view* at the SI joint (diameter *1*), in the middle of the distance between SI joint and midsagittal plane (diameter *2*) and in the midsagittal plane (diameter *3*)

True inlet, outlet, and lateral views of the sacrum were obtained by using a two-dimensional reconstruction tool in a standardized and reproducible manner. In a first step, the midsagittal plane was selected in the lateral view (Fig. 1a). The axes were adjusted parallel to the lower endplate of S1 (red lines) and along the posterior boarder of S1 (blue line). In a next step, both axes were adjusted in the outlet view (Fig. 1b) to the vertical axis of the sacrum (green line) and parallel to a line connecting the cranial borders of the SI joint (red line). In a last step, the adjustment in the inlet view (Fig. 1c) included alignment in a sagittal axis from the middle of the promontorium to the spinous process (green line) and in the coronal plane parallel to posterior wall of the body of S1 (blue line). The same procedure was performed in S2 as well.

The assessment of the width of the corridor in the inlet view (inlet corridor) is shown in Fig. 1d. A line parallel the posterior wall of S1 (blue line) served as a reference for the measurement. The width of the corridor was defined as the perpendicular distance between the anterior cortex of the sacrum and this line. The corridor was assessed on both sides at the SI joint (diameter 1), in the middle of the distance between SI joint and midsagittal plane (diameter 2) and in the midsagittal plane (diameter 3) on the right side and left side. In addition, the corridor was assessed in the upper quartile, in the middle and in the lower quartile of the craniocaudal extension of the body S1 by parallel translation of the red axis in the sagittal view (Fig. 1a). The same procedure was performed in S2 as well.

**Fig. 2:**
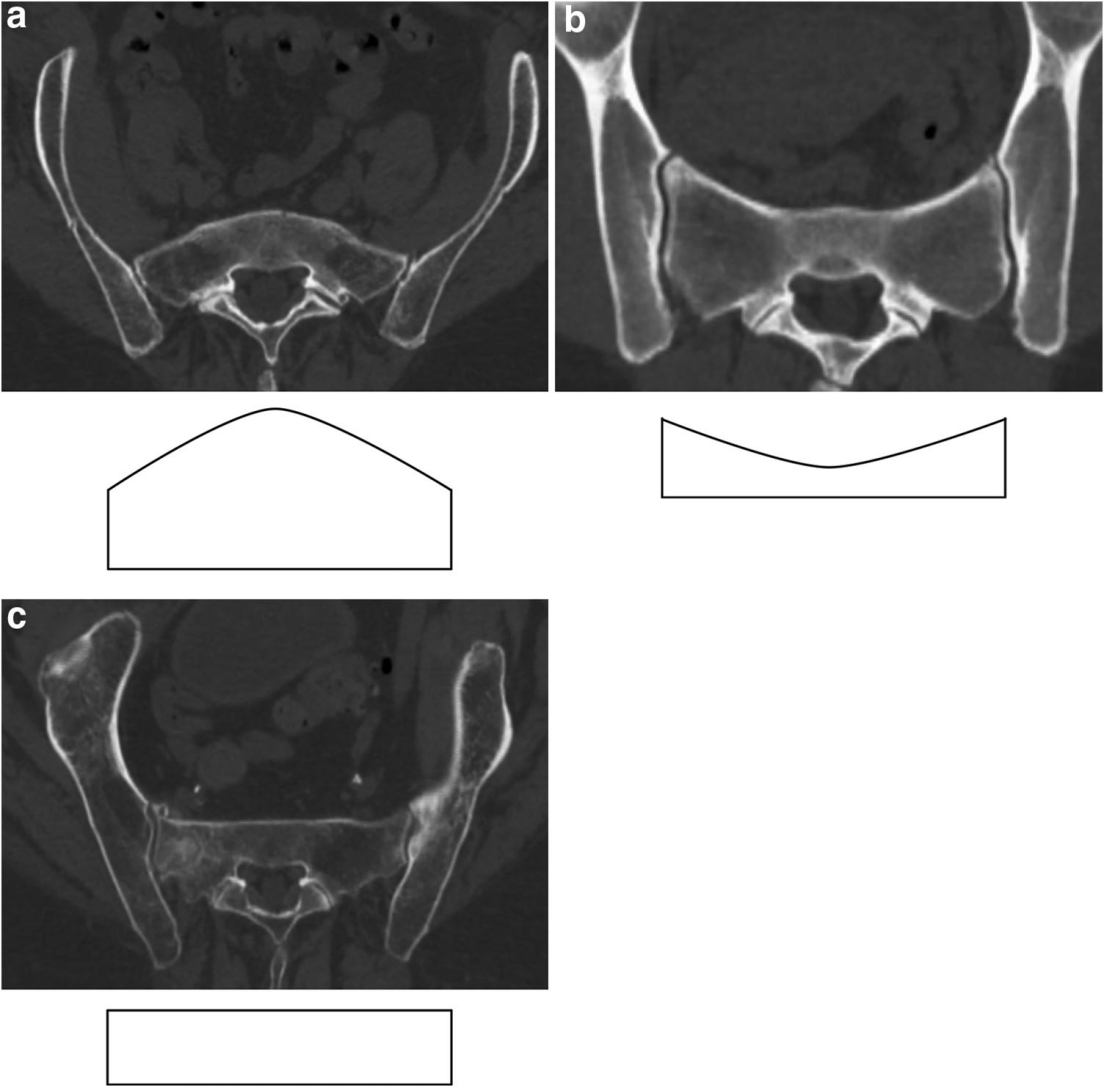
**a** Ascending type. **b** Descending type. **c** Horizontal type

Subsequently, the diameters were used to calculate different types of inlet corridors. In a first step, the corridors were either defined as ascending, horizontal, or descending corridors. In an ascending corridor, the diameter 1 (SI joint) is smaller than the diameter 3 (midsagittal, Fig. 2a). In a descending corridor, the diameter 1 is larger than the diameter 3 (Fig. 2b). In a horizontal corridor, the diameters 1 and 3 are equal (Fig. 2c). A threshold of 3 mm was applied to eliminate marginal differences between the diameters, which are not clinically relevant. Accordingly, the diameter 3 in an ascending corridor should be minimum more than 3 mm larger than the diameter 1 (and vice versa in a descending corridor).

**Fig. 3:**
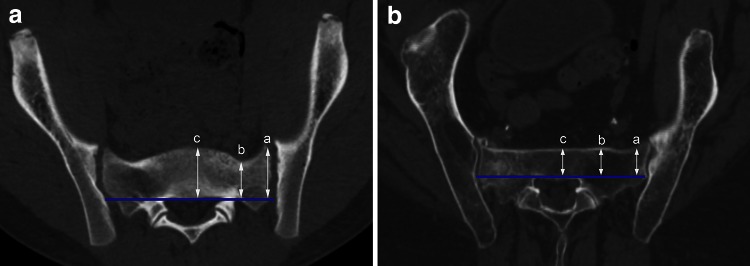
**a** Horizontal type with notch. **b** Horizontal type without notch

In a second step, the presence of a ‘‘notch’’ was assessed. A notch is an indentation of the anterior cortex of the sacrum between the SI joint and the midsagittal plane with proximity to the nerve root L5, which, therefore, may be harmed during screw misplacement. The arithmetic mean between diameter 1 and 3 was calculated, which is equivalent to diameter 2 in the presence of a straight-lined anterior cortex between SI joint and the midsagittal plane. If the measured diameter 2 is more than 3 mm smaller than the calculated diameter 2, the presence of a notch was defined (Fig. 3a). Figure 3b shows an example of a horizontal corridor without notch.

SPSS Statistics 21.0 (SPSS, Chicago, IL, USA) was used for the statistical analysis. Metric scaled data are reported as arithmetic mean ± standard deviation and categorical data as absolute frequency and percentage distribution. Correlations for metric scaled data were quantified using the Pearson coefficient and for non-parametric samples using the Spearman coefficient. The Kolmogorov–Smirnov test was used for the determination of the distribution form. The probability level was set at *p* < 0.05.

## Results

CT scans of 1000 patients were analyzed in this study. Measurements were performed both on the right side and left side resulting in 2000 measurements. The mean age of the patients was 68.8 ± 21.5 years. There were 623 female (62.2 %) and 387 male patients (37.8 %) included.

The results of the assessment of the different corridor types are shown in Table 1. While the ascending type was the most frequent corridor type in the upper quartile of S1 (71 %), its relative frequency declined to 5 % in the lower quartile. There were no ascending types in S2. Descending types were found in 15 % in the upper quartile of S1 and in 86 % in the lower quartile. In S2, descending types were, by far, the most frequent corridor type. Horizontal types were less frequent with a relative incidence between 2 and 14 % (Table 1). The results of the assessment of the presence of a notch are shown in Table 2. We found a notch in the majority of cases with relative frequencies ranging from 69 % (upper quartile of S1) to 95 % (upper quartile of S2) (Table 2). The mean depths of the notch are shown in Table 3. There were no significant differences between the different groups.

**Table 1:** Distribution of the relative frequency of the different corridor types in the upper, middle, and lower quartiles of S1 and S2

	S1 upper quartile (%)	S1 middle quartile (%)	S1 lower quartile (%)	S2 upper quartile (%)	S2 middle quartile (%)	S2 lower quartile (%)
Ascending type	71	21	5	0	0	0
Horizontal type	14	12	9	2	2	5
Descending type	15	66	86	98	98	95

**Table 2:** Distribution of the relative frequency of the presence of a notch in the upper, middle, and lower quartiles of S1 and S2

	S1 upper quartile	S1 middle quartile	S1 lower quartile	S2 upper quartile	S2 middle quartile	S2 lower quartile
Number (*n* = 2000)	1380	1495	1878	1897	1604	1711
Percent	69 %	75 %	94 %	95 %	80 %	86 %

**Table 3:** Depth of the notch in the upper, middle, and lower quartiles of S1 and S2 (in mm)

	Mean (±SD)
S1 upper quartile	9.9 (±7.0)
S1 middle quartile	8.5 (±5.2)
S1 lower quartile	9.4 (±4.3)
S2 upper quartile	11.8 (±7.6)
S2 middle quartile	6.1 (±2.3)
S2 lower quartile	7.5 (±5.1)

## Discussion

Percutaneous SI screw fixation under fluoroscopic control is an effective and widely used method for the stabilization of the posterior pelvic ring. Screw misplacement with consecutive nerve lesions, mainly of the nerve root L5, is a major complication of this technique [6, 16–20]. Interindividual variations in the morphology of the upper sacrum, the so-called sacral dysmorphism, particularly account for these findings. Sacral dysmorphism has been described by several authors. Most of these studies, however, are anatomical studies describing the morphological variance of the upper sacrum without assessing their relevance for percutaneous SI screw fixation [26–28]. Other authors assess the sacral morphology considering its clinical relevance for SI screw fixation. Carlson, for example, described the three-dimensional shape of the corridor for SI screw fixation (vestibule concept) in a CT-based study, [23], while Mendel showed a CT-based 3D model of secure bone corridors (safe zones) and optimal trajectories for sacroiliac screws [24]. The authors, additionally, provided recommendations for the angulation of SI screws in different planes. These recommendations, however, differ significantly between the authors, which, obviously, is not surprising given the variations in sacral morphology. The angle in a caudocranial direction (outlet view), for example, ranged from 2° to 18°, while the angle in a posteroanterior direction (inlet view) ranged from 2° to 16° [23, 24]. In conclusion, a ‘‘one-size-fits-all’’ approach to percutaneous SI screw fixation is not feasible given the diversity of the sacral morphology.

To avoid screw misplacement in percutaneous SI screw fixation, CT guided and navigated procedures have been developed [9–12, 14, 15, 29]. These procedures allow for secure screw placement even in the presence of narrow osseous corridors and sacral dysmorphia, which was demonstrated in the above-mentioned studies. Besides a higher radiation exposure [14], the major drawback of these techniques, however, is their limited availability beyond trauma centers. Accordingly, SI screw fixation under fluoroscopic control is and will continue to be an important technique for minimally invasive stabilization of the posterior pelvic ring.

SI screw fixation under fluoroscopic control not only relies on sound intra-operative imaging, but also on the surgeon´s ability to interpret the two-dimensional images in a 3D context. Several authors have provided different approaches for the interpretation of these images in an inlet, outlet, and lateral views [4, 30–32]. Routt, for example, reported that abnormal morphological patterns of the upper sacrum could be easily identified using mainly the outlet view and the lateral view [30]. We agree that the outlet view allows for secure screw placement above the first sacral foramina in S1 even in the presence of dysmorphic foramina. The lateral view allows for the assessment of the upper, lower, and posterior borders of the osseous corridor. However, we disagree that the lateral view is valuable for the assessment of the anterior border of the corridor. First, the anterior border is not strict parallel to the projection in the lateral view. Our data show that the anterior border may be divergent (ascending type), horizontal, or convergent (descending type) relative to the posterior border, which highly influences its radiological appearance in the lateral view. Second, indentations of the anterior cortical border of the corridor (notches) may not be assessable in a view axial to this border. The techniques of corridor assessment described by Mendel [31] and Noojin [32] neither assess the ‘‘notch’’ problem as well.

Miller [33] summarized the three key points of fluoroscopically controlled SI screw fixation in the presence of sacral dysmorphism in a recent review article as follows: First, dysmorphic sacral neuroforamina are assessed in the outlet view. Second, the posterior wall and variances in the sacral alar slope are assessed in the lateral view. Third, notches of the anterior cortex are assessed in the inlet view. According to our own experience, however, the inlet view may not be appropriate to reliably detect notches of the anterior cortex in all cases. The inlet view is a two-dimensional projection with an overlap of the anterior cortex of the vertebral bodies S1 and S2. Our data, however, show that the anterior cortex of the upper sacrum is not a flat surface in a three-dimensional space, which would result in a single line in a two-dimensional projection. The corridor type, for example, changes very frequently within the vertebral body of S1 (Table 1). In addition, the presence or absence of a notch is not detectable in an inlet view in the majority of cases and further limits the assessment of the anterior cortex of the upper sacrum in this view.

The fluoroscopic control during SI screw fixation is adequate when drilling in a strict transverse direction. Transverse corridors of adequate width, however, are present in a minority of patients only. Ascending corridors with a notch have a very narrow intraosseous corridor in a transverse direction. These types require a more posterior entry point (even posterior to the posterior wall of S1 in a lateral view) and a more ascending drilling direction, which increases the risk of nerve lesions particularly in the additional presence of a notch. The aim of our study, therefore, was to develop a simple method for the preoperative assessment of the corridor type and the presence of a notch with widely available tools as shown above. We consider horizontal and descending corridor types without a notch to be feasible for fluoroscopically controlled SI screw fixation, while ascending types with notch bear a higher risk of iatrogenic nerve lesions. In these cases, we recommend navigated SI screw fixation. One interesting finding of our study was the high frequency of ascending corridors in the upper quartile of S1 with a much lower frequency in the middle quartile and lower quartile of S1. An ascending corridor in the upper quartile is mainly determined by the inclusion of the sacral promontory in the corridor analysis. In the upper quartile, there was an additional notch in 69 % of the cases as well. Accordingly, we consider a screw trajectory to the promontory to be associated with a higher risk of L5 nerve root lesions than more flat screw trajectories.

One limitation of our study has to be noted. It was our goal to classify the morphology of the upper sacrum based on the risk of iatrogenic nerve lesions during percutaneous SI screw fixation. Accordingly, we focused on the corridor morphology in the inlet view and defined different inlet corridor types as well as the presence of a notch. For a safe overall screw placement additional parameters, such as the absolute diameter of the corridor in mm and corridors in the outlet view are relevant as well. The assessment of these parameters, however, was not the goal of this study.

In conclusion, our concept obviously is not a “one-size-fits-all’’ concept neither. It aims at three goals: (a) to increase the awareness of the surgeon for the problem of sacral dysmorphism, (b) to emphasize the importance of a preoperative assessment of the osseous corridor, and (c) to provide a simple method for the preoperative assessment with widely available tools.
